# The incidence and influencing factors of hypertensive disorders during pregnancy in hangzhou, China, between 2012 and 2021

**DOI:** 10.1016/j.heliyon.2025.e41680

**Published:** 2025-01-08

**Authors:** Yiming Chen, Bin Wu, Huimin Zhang, Xuelian Chu, Lingling Huang, Yanan Wang, Xiufeng Liang

**Affiliations:** a Prenatal Diagnosis and Screening Center, Hangzhou Women's Hospital (Hangzhou Maternity and Child Health Care Hospital), Hangzhou, Zhejiang, 310008, China; b The Fourth School of Clinical Medical, Zhejiang Chinese Medical University, Hangzhou, Zhejiang, 310053, China; c The First People's Hospital of Yuhang District, Hangzhou, Zhejiang, 311113, China; d Department of Laboratory, Maternal and Child Health Hospital of Linping District, Hangzhou, Zhejiang, 311100, China; e Department of Laboratory, The Affiliated Hospital of Hangzhou Normal University, Hangzhou, Zhejiang, 310015, China

**Keywords:** Hypertensive disorders of pregnancy, Incidence rate, Risk factors, Gestational hypertension, Preeclampsia, Severe preeclampsia, Maternal age, Odds ratios, Pregnancy outcomes, Retrospective cohort study

## Abstract

**Objective:**

To analyze the incidence and influencing factors of hypertensive disorders of pregnancy (HDP) in Hangzhou, China, between 2012 and 2021.

**Methods:**

We conducted a retrospective cohort study to analyze data relating to 155,433 pregnant women in Hangzhou, China, who were divided into eight groups: gestational hypertension (GH, 4247 cases), preeclampsia (PE, 1602 cases), severe preeclampsia (SPE, 995 cases), eclampsia (9 cases), HELLP syndrome (43 cases), chronic hypertension (43 cases), chronic hypertension combined with PE or SPE (77 cases), postpartum eclampsia (5 cases), and pregnant women with normal blood pressure (148412 cases). Odds ratios (ORs) and 95 % confidence intervals (95 % CIs) were used to show the association of factors with HDP.

**Results:**

The total incidence of HDP was 45.17 ‰ (95 % CI: 44.14‰–46.20 ‰), and the subtypes of HDP showed an increasing trend from 2012 to 2021 (all *P* < 0.01). Several maternal characteristics, including maternal age (OR_GH_ = 1.158 (1.035–1.295), 1.697 (1.478–1.948), and 2.769 (2.234–3.433), OR_PE_ = 1.526 (1.224–1.902) to 2.506 (1.796–3.497)), non-local residence (OR = 1.124 (1.018–1.241), 1.212 (1.032–1.423), and 1.222 (1.010–1.479)), *in vitro* fertilization (OR = 1.263 (1.028–1.551), 1.342 (1.042–1.729) and 1.692 (1.234–2.321)), graviditas ≤1 (OR_GH_ = 1.126 (1.035–1.226)), parity <1 (OR_GH_ = 1.140 (1.026–1.265), OR_PE_ = 1.336 (1.130–1.581)), non-vaginal delivery (OR_GH_ = 1.525 (1.223–1.901), 1.185 (1.058–1.328) an 1.753 (1.612–1.908); OR_pe_ = 2.108 (1.428–3.112), 1.464 (1.174–1.826) and 4.205 (3.643–4.854), and OR_spe_ = 7.000 (3.517–13.930), 1.914 (1.217–3.010) and 19.981 (14.936–26.730)), maternal hospitalization >7 days (OR = 2.098 (1.921–2.291), 2.115 (1.857–2.409), and 1.553 (1.333–1.810)), and winter birth (OR_GH_ = 1.297 (1.196–1.408), OR_PE_ = 1.425 (1.242–1.636)), were all identified as risk factors for GH, PE, and SPE. Furthermore, HDP may increase the risk of gestational period <37 weeks, low birth weight (LBW), fetal macrosomia, and a birth length <50 cm (all *P* < 0.05).

**Conclusions:**

The incidence of HDP and its subtypes showed an overall increasing trend from 2012 to 2021. Maternal factors and pregnancy outcomes may be strongly associated with HDP.

## Highlights

• The etiology and pathogenesis of HDP have not been clarified.
• The incidence of HDP and its subtypes showed an overall increasing trend.
• Maternal factors and pregnancy outcomes may be strongly associated with HDP.

## 1 Introduction

Hypertensive disorders of pregnancy (HDP), including gestational hypertension (GH), preeclampsia (PE), severe preeclampsia (SPE), and eclampsia, are the main causes of perinatal adverse outcomes [[1], [2], [3]]. However, the precise etiology and pathogenesis of HDP have yet to be elucidated. PE is a symptom of hypertension, proteinuria, other multi-system involvement, and target organ damage after 20 weeks of gestation, and is the most representative type of HDP [[4], [5], [6], [7]]. Complications related to PE can lead to the early induction of labor or cesarean section and subsequent preterm birth. The US Preventive Services Task Force Recommendation Statement (USPSTF) recommends blood pressure measurement throughout pregnancy to screen for maternal PE [8].

HDP has become one of the most important challenges facing public health, with the prevalence of HDP, GH, and PE reaching 5.20–8.20 %, 1.80–4.40 %, and 0.20–9.20 %, respectively. Body mass index (BMI), anemia, and low educational attainment appear to be modifiable risk factors for HDP. Advanced maternal age (AMA), first birth, multiple pregnancy, a history of HDP pregnancy, a previous history of hypertension, a previous history of type 2 diabetes, a previous family history of urinary tract infection and hypertension, gestational diabetes, type 2 diabetes, and PE, appear to be unmodifiable risk factors [9]. PE is closely associated with acute maternal morbidity, stillbirth, cesarean section, and preterm birth [10]. However, a previous study reported that the prevalence and risk of PE decreased despite an increase in the proportion of high-risk women, including AMA, and those undergoing premature delivery and assisted reproduction. These findings may be related to an increase in the use of aspirin preventive therapy among childbearing women, an overall increase in induced labor, a lower mean blood pressure in pregnant women, and an improvement in population health status [11]. This suggests that clinical intervention may partially explain the observed reduction in the prevalence of PE.

To investigate the prevalence of HDP and its subtypes in Hangzhou, China, we investigated the prevalence of HDP, and its subtypes, in a cohort of 155,433 women who delivered in the Obstetric Departments of three hospitals in Hangzhou, China, between March 2012 and February 2022. We evaluated the trend for prevalence during this study period and investigated whether disease prevalence was influenced by a range of risk factors.

## 2 Materials and methods

### 2.1 Subjects

**Fig. 1:**
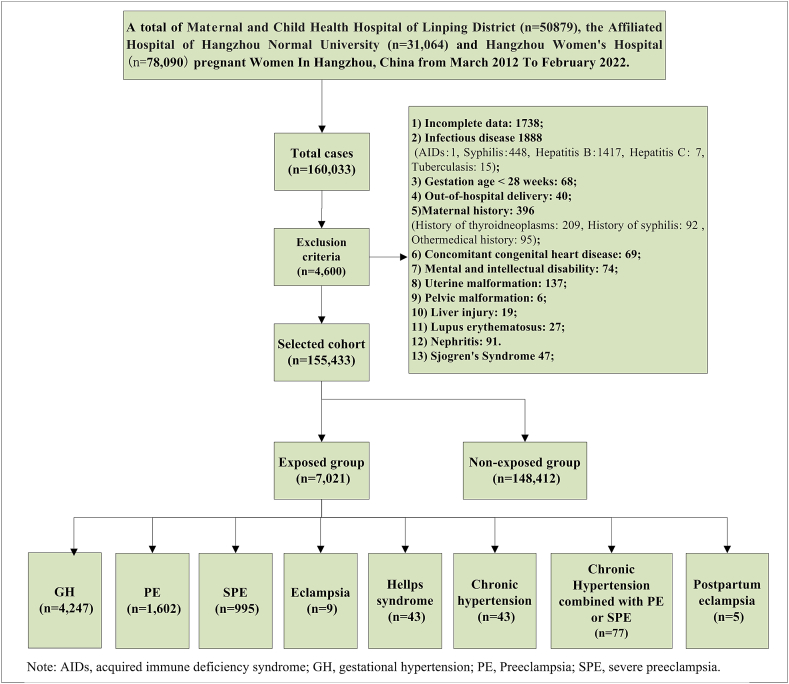
Flowchart of groups among 155,433 pregnant women with hypertensive disorders during pregnancy and its subtypes.

In this retrospective cohort study, we collected 160,033 data from the perinatal electronic medical records databases relating to in-patient delivery in the Obstetrics and Gynecology Departments of three tertiary hospitals in Hangzhou, China, between March 2012 and February 2022 in Maternal and Child Health Hospital of Linping District, Hangzhou (n=50,879), and the Affiliated Hospital of Hangzhou Normal University (n=31,064) and from November 2014 to February 2022 in Hangzhou Women's Hospital (n=78,090), respectively. Each of these databases features information recorded by obstetricians immediately after each delivery, including the International Classification of Diseases (ICD) code. In total, there were 4247 cases of GH, 1602 cases of PE, 995 cases of SPE, nine cases of eclampsia, 43 cases of HELLP syndrome, 43 cases of chronic hypertension, 77 cases of chronic hypertension combined with PE or SPE, five cases of postpartum eclampsia, and 148,412 cases with normal blood pressure. Thus, our study cohort included a total of 155,433 pregnant women, as shown in Fig. 1. This study was discussed and approved by the Hangzhou Women's Hospital Medical Ethics Committee 2023-A-(013). Since this study was retrospective, and the data were processed anonymously, the Hospital Ethics Committee approved the exemption of patient informed consent.

### 2.2 Diagnostic and exclusion criteria

#### 2.2.1 Diagnostic criteria

For each case, a diagnosis was made according to the HDP Diagnostic Guidelines (2015) [12], in line with the 10th Clinical Revision of the International Classification of Diseases (ICD; ICD-10-CM) for GH, PE, SPE, and comorbidities. GH was diagnosed as hypertension occurring for the first time after 20 weeks of pregnancy, with a systolic blood pressure ≥140 mmHg (1 mmHg = 0.133 kPa) and/or a diastolic blood pressure ≥90 mmHg, returning to normal within 12 weeks postpartum, with a negative test for protein in the urine. PE was diagnosed by a systolic blood pressure ≥140 mmHg and/or a diastolic blood pressure ≥90 mmHg after 20 weeks of gestation, accompanied by any of the following: urinary protein ≥0.30 g/24 h, or urinary protein/creatinine ratio ≥0.30, or random urinary protein ≥ (+); no proteinuria, but accompanied by heart, lung, liver, kidney and other important organs, or abnormal changes in the blood system, digestive system, nervous system, placenta, fetal involvement, or the involvement of any organ or system. PE can be classified as mild or severe depending upon disease progression. Chronic hypertension (CH) refers to high blood pressure diagnosed prior to pregnancy, 20 weeks of gestation, or 6 weeks postpartum (with a systolic blood pressure >140 mmHg and/or a diastolic blood pressure >90 mmHg). Eclampsia is a newly occurring tonic-clonic, focal, or multifocal seizure, or in the absence of other causes, an unexplained change in mental status in pregnant or postpartum patients.

#### 2.2.2 Inclusion criteria

We included pregnant women and newborns who gave birth in the three designated Hospitals between March 2012 and February 2022 with a gestational age ≥28 weeks.

#### 2.2.3 Exclusion criteria

We excluded patients with a gestation age <28 weeks, those undergoing home birth, or those with infectious diseases such as AIDS, syphilis, hepatitis B, hepatitis C and tuberculosis. We also excluded patients with chronic hypertension, heart disease, kidney disease, diabetes, thyroid tumors, hyperthyroidism, connective tissue disease, blood disease, and other chronic histories. We also excluded those who smoked, those for whom the outcome of pregnancy included chromosomal abnormalities or other birth defects, those with a history of immunotherapy and blood transfusion, a history of special medication during pregnancy, and mental retardation. We also excluded cases with an incomplete set of clinical information.

#### 2.2.4 Definitions of pregnancy complications and pregnancy outcomes

Pregnancy complications include HDP, gestational diabetes mellitus (GDM), intrahepatic cholestasis of pregnancy (ICP), hyperlipidemia, anemia in pregnancy, Placenta previa, abnormal thyroid function, intrauterine growth retardation, and oligohydramnios [[13], [14], [15]]. Pregnancy combined with pathogenic infection refers to hepatitis B virus carriers, mycoplasma, chlamydia, streptococcus, and other forms of pathogenic infection [[16], [17], [18]]. Pregnancy outcomes included preterm birth, postpartum hemorrhage, fetal distress, cesarean section, low birth weight, and macrosomia.

ICP was diagnosed by marked skin pruritus and varying degrees of jaundice during pregnancy, mild or moderate elevation of bile acids and total bilirubin upon laboratory examination, and their disappearance upon termination of pregnancy. Placenta previa is a condition in which the placenta remains attached to the lower part of the uterus after 28 weeks of pregnancy, and the lower edge of the placenta reaches or covers the inner opening of the cervix, at a position lower than that of the fetus. Anemia during pregnancy refers to a hemoglobin concentration <11 g/dL during pregnancy. Intrauterine growth retardation was defined as an estimated fetal weight < adjusted for gestational age [M (P10)] on ultrasound during pregnancy. Oligohydramnios refers to an amniotic fluid index (AFI) < 5 cm, and polyhydramnios refers to an AFI ≥25 cm. Preterm birth refers to a birth that occurs prior to 37 weeks of gestation. Postpartum hemorrhage refers to a blood loss ≥500 mL after vaginal delivery or ≥1000 mL following cesarean section. Fetal distress refers to the fact that the fetus has signs of hypoxia *in utero*, which can endanger the health and life of the fetus. Cesarean section refers to an operation through the abdomen to remove the fetus. Low birth weight (LBW) refers to a birth weight <2500 g within 1 h after birth, while macrosomia refers to a birth weight >4000 g within 1 h of birth. All pregnancy complications and pregnancy outcomes were obtained from clinical records diagnosed by hospital obstetricians according to Chinese guidelines.

### 2.3 Statistical analysis

IBM-SPSS version 21.0 statistical software (IBM Corp. Released 2012. IBM SPSS Statistics for Windows, Version 21.0. Armonk, NY: IBM Corp.) was used for statistical processing. A one-sample Kolmogorov-Smirnov test was used to test data for normality. Data that did not confirm to a normal distribution are represented by median and percentile [M (P_2.5_, P_97.5_)] and comparisons between two or more groups were performed using the Mann-Whitney U or Mann-Whitney H test. Pearson Chi-squared tests or adjusted for Chi-squared test were used to compare single factors for different maternal characteristics with the two experimental groups, and *P* < 0.10 was used as a criterion for inclusion in subsequent multivariate logistic regression analysis. Finally, multiple logistic regression analysis was performed to analyze maternal age, non-local residence, uterine scar history following cesarean section, multiple pregnancy, *in vitro* fertilization, pregnancy complications, graviditas, parity, delivery hospitals, methods of delivery, hospital days, birth year, birth season, gestational week, birth weight, fetal length, and fetal abnormalities as covariables. In addition to 6844 cases with a high incidence of GH, PE and SPE, the incidence of the other five subtypes of HDP was very low; thus, it was not appropriate to apply multiple logistic regression analysis for these five HDP subtypes. Multivariate logistic regression analysis was performed to screen the odds ratio (OR) and 95 % (confidence interval, CI) of relevant influencing factors. *P* < 0.05 was considered to be statistically significant.

## 3 Results

### 3.1 Overall incidence of HDP

**Fig. 2:**
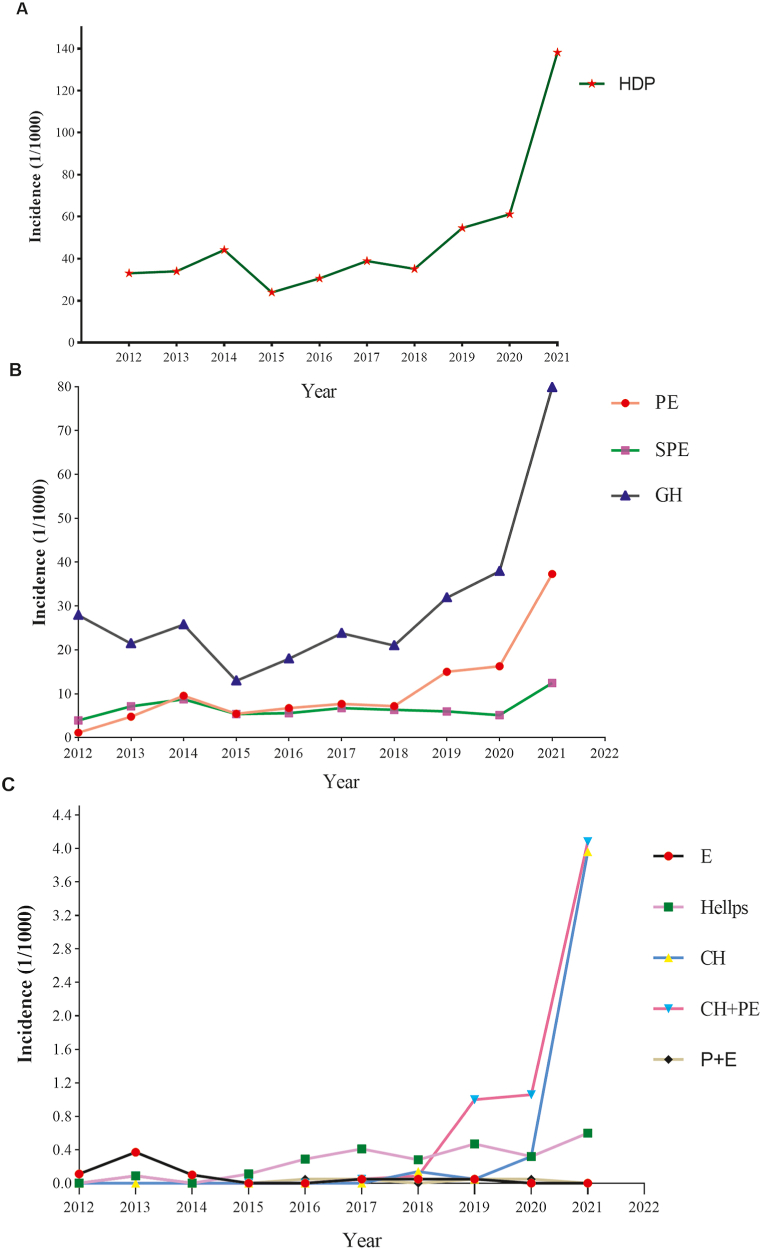
The incidence of sub-classes of hypertensive disorders during pregnancy in Hangzhou, China, 2015–2021. A, The incidence of HDP; B, The incidence of GH, PE and SPE; C. The incidence of other sub-classes of HDP. HDP, Hypertensive disorders of pregnancy; CH, Chronic hypertension; GH, gestational hypertension; PE, Preeclampsia; SPE, severe preeclampsia; E, eclampsia; Hellps, HELLP syndrome; CH+PE, chronic hypertension combined with PE or SPE; P+E, postpartum eclampsia.

Between 2012 and 2021, 155,433 women with a gestation age >28 weeks gave birth in the hospital; 7021 cases were diagnosed with HDP following discharge, with a total incidence of 45.17 ‰ (95 % CI: 44.14‰–46.20 ‰), including 4247 cases of GH, 1602 cases of PE, 995 cases of SPE, nine cases of eclampsia, 43 cases of HELLP syndrome and 43 cases of chronic hypertension. There were 77 cases of chronic hypertension combined with PE or SPE and five cases of postpartum eclampsia; the incidence rates were 27.32 ‰ (95 % CI: 26.51‰–28.13 ‰), 10.31 ‰ (95 % CI: 9.81 ‰–10.81 ‰), 6.40 ‰ (95 % CI: 6.01 ‰–6.80 ‰), 0.06 ‰ (95 % CI: 0.02‰–0.10 ‰), 0.28 ‰ (95 % CI: 0.20‰–0.36 ‰), 0.28 ‰ (95 % CI: 0.20‰–0.36 ‰), 0.50 ‰ (95 % CI: 0.39 ‰–0.61 ‰) and 0.03 ‰ (95 % CI: 0.00–0.06 ‰), respectively, as shown in Fig. 2.

### 3.2 The trend for HDP incidence between 2012 and 2021

There were significant differences in the incidence of HDP across different years (*P* < 0.01 for all). Chi-squared tests revealed that the incidence of HDP increased between 2012 and 2021; all types of HDP showed an increasing trend (all *P* < 0.01), as shown in Fig. 2A.

The incidence of each subtype of HDP showed an upwards trend from 2012 (all *P* < 0.01), except for GH, which showed a downwards trend from 2012 to 2015, followed by an upwards trend annually from 2016, as shown in Fig. 2B.

### 3.3 Analysis of influencing factors for GH, PE and SPE

In total, there were 6844 patients with HDP (GH, n = 4247; PE, n = 1602; SPE, n = 995) compared with 148,412 non-HDP parturients. We identified statistically significant differences between the two groups in terms of maternal age, non-local residence, uterine history of scarring after cesarean section, multiple pregnancy, *in vitro* fertilization, pregnancy complications, graviditas, parity, delivery hospitals, methods of midwifery, hospital days, birth year, birth season, gestational weeks, birth weight, fetal length and the structural proportions of fetal abnormalities (all *P* < 0.10). However, there was no statistically significant difference between fetal sexes (all *P* > 0.10), as shown in Table 1, Table 2.

**Table 1:** Univariate analysis of the demographic of four groups of pregnant women n (%).

Indicators	Groups				*Z/x*^*2*^	*P value*
	GH (n = 2614)	PE (n = 1602)	SPE (n = 995)	non-HDP (n = 148412)		
Maternal age (M (P_2.5_, P_97.5_) years)	29.00 (21.00–40.00)	29.00 (21.00–40.00)	29.00 (20.00–40.00)	28.00 (21.00–38.00)	154.899	<0.001
Maternal age group (years)					323.154	<0.001
< 20	57 (1.3)	20 (1.2)	19 (1.9)	1931 (1.3)		
20–24.9	558 (13.1)	208 (13.0)	154 (15.5)	22859 (15.4)		
25–29.9	1683 (39.6)	650 (40.6)	369 (37.1)	65520 (44.1)		
30–34.9	1313 (30.9)	474 (29.6)	301 (30.3)	43830 (29.5)		
35–39.9	514 (12.1)	199 (12.4)	124 (12.5)	12471 (8.4)		
≥ 40	122 (2.9)	51 (3.2)	28 (2.8)	1801 (1.2)		
Residence					34.370	<0.001
non-local	1476 (34.8)	594 (37.1)	438 (44.0)	56335 (38.0)		
local	2771 (65.2)	1008 (62.9)	557 (56.0)	92077 (62.0)		
Multiple pregnancy					573.493	<0.001
Yes	70 (1.6)	96 (6.0)	73 (7.3)	1779 (1.2)		
No	4177 (98.4)	1506 (94.0)	922 (92.7)	146633 (98.8)		
*In vitro* fertilization					403.029	<0.001
Yes	114 (2.7)	88 (5.5)	57 (5.7)	1886 (1.3)		
No	4133 (97.3)	1514 (94.5)	938 (94.3)	146526 (98.7)		
Uterine scar					11.305	0.010
Yes	686 (16.2)	233 (14.5)	156 (15.7)	25402 (17.1)		
No	3561 (83.8)	1369 (85.5)	839 (84.3)	123010 (82.9)		
Complication of pregnancy					68.739	<0.001
Yes	2170 (51.1)	888 (55.4)	560 (56.3)	71684 (48.3)		
No	2077 (48.9)	714 (44.6)	435 (43.7)	76728 (51.7)		
Graviditas					76.995	<0.001
No (1)	1891 (44.5)	753 (47.0)	417 (41.9)	58852 (39.7)		
Yes (≥2)	2356 (55.5)	849 (53.0)	578 (58.1)	89560 (60.3)		
Parity					146.144	<0.001
No (0)	2677 (63.0)	1094 (68.3)	626 (62.9)	84923 (57.2)		
Yes (≥1)	1570 (37.0)	508 (31.7)	369 (37.1)	63489 (42.8)		
Gestational age (M (P_2.5_, P_97.5_) weeks)	38 (35–41)	38 (33–40)	37 (30–40)	39 (35–41)	1754.854	<0.001
Gestational age groups (weeks)					3027.100	<0.001
< 37	271 (6.4)	280 (17.5)	418 (42.0)	7873 (5.3)		
> 41	149 (3.5)	39 (2.4)	17 (1.7)	10567 (7.1)		
37-41	3827 (90.1)	1283 (80.1)	560 (56.3)	129972 (87.6)		
Mode of delivery					1850.128	<0.001
Forceps delivery	92 (2.2)	29 (1.8)	10 (1.0)	2335 (1.6)		
Lateral vaginal incision	456 (10.7)	116 (7.2)	31 (3.1)	17128 (11.5)		
Anterior vaginal incision	141 (3.3)	26 (1.6)	6 (0.6)	5328 (3.6)		
Cesarean section	2184 (51.4)	1117 (69.7)	895 (89.9)	59281 (39.9)		
Vaginal delivery	1374 (32.4)	314 (19.6)	53 (5.3)	64340 (43.4)		
Birth Years groups (years)					521.850	<0.001
2012	250 (5.9)	10 (0.6)	35 (3.5)	8037 (5.4)		
2013	232 (5.6)	52 (3.2)	77 (7.7)	8588 (5.8)		
2014	259 (6.1)	96 (6.0)	88 (8.8)	10377 (7.0)		
2015	227 (5.3)	95 (5.9)	94 (9.4)	9636 (6.5)		
2016	368 (8.7)	138 (8.6)	115 (11.6)	16869 (11.4)		
2017	469 (11.0)	152 (9.5)	133 (13.4)	19740 (13.3)		
2018	456 (10.7)	156 (9.7)	138 (13.9)	18941 (12.8)		
2019	606 (14.3)	285 (17.8)	114 (11.5)	20700 (13.9)		
2020	715 (16.8)	307 (19.2)	97 (9.7)	17830 (12.0)		
2021	665 (15.7)	311 (19.4)	104 (10.5)	17694 (11.9)		
Birth season					379.864	<0.001
Spring	1019 (24.0)	345 (21.5)	209 (21.0)	33181 (22.4)		
Summer	769 (18.1)	312 (19.5)	237 (23.8)	37105 (25.0)		
Autumn	981 (23.1)	408 (25.5)	286 (28.7)	41946 (28.3)		
Winter	1478 (34.8)	537 (33.5)	263 (26.4)	36180 (24.4)		
hospital of delivery					300.687	<0.001
hospital 1	2309 (54.4)	854 (53.3)	444 (44.6)	72317 (48.7)		
hospital 2	982 (23.1)	432 (27.0)	225 (22.6)	47728 (32.2)		
hospital 3	956 (22.5)	316 (19.7)	326 (32.8)	28367 (19.1)		
Maternal hospital stay (M (P_2.5_, P_97.5_) days)	5 (3–13)	6 (3–15)	6 (4–18)	5 (3–11)	2408.101	<0.001
Maternal hospital stay groups (days)					1417.973	<0.001
>7	707 (16.6)	350 (21.8)	293 (29.4)	11519 (7.8)		
≤ 7	3540 (83.4)	1252 (78.2)	702 (70.6)	136893 (92.2)		

Note: GH: gestational hypertension; PE: preeclampsia; SPE: severe preeclampsia; HDP: Hypertensive disorders of pregnancy.

**Table 2:** Univariate analysis of neonatal demographic of four groups n (%).

Indicators	Groups				*Z/x*^*2*^	*P value*
	GH (n = 2614)	PE (n = 1602)	SPE (n = 995)	non-HDP (n = 148412)		
Birth weight (M (P_2.5_, P_97.5_) g)	3240 (2220–4198)	3125 (1750–4269)	2735 (1129–4158)	3300 (2400–4150)	964.117	<0.001
Birth weight groups (g)					4438.170	<0.001
<2500	230 (5.4)	251 (15.7)	393 (39.7)	4823 (3.2)		
>4000	189 (4.5)	94 (5.9)	32 (3.2)	6155 (4.1)		
2500-4000	3828 (90.1)	1257 (78.5)	568 (57.1)	137434 (92.6)		
Birth length (M (P_2.5_, P_97.5_) cm)	50 (47–52)	50 (43–52)	49 (36–52)	50 (47–52)	1227.886	<0.001
Birth length groups (cm)					1691.885	<0.001
<50	725 (17.1)	439 (27.4)	541 (54.4)	19754 (13.3)		
≥50	3522 (82.9)	1163 (72.6)	454 (45.6)	128658 (86.7)		
Apgar score (M (P_2.5_, P_97.5_))	10 (9.3–10)	10 (8.58–10)	10 (6.5–10)	10 (9.5–10)	616.061	<0.001
Apgar score groups					607.266	<0.001
<10	126 (4.7)	82 (8.4)	141 (24.8)	3510 (4.2)		
≥10	2536 (95.3)	895 (91.6)	427 (75.2)	78957 (95.8)		
Newborn gender					11.343	0.666
male	2236 (52.6)	858 (53.6)	508 (51.1)	77961 (52.5)		
female	2011 (47.4)	744 (46.4)	487 (48.9)	70451 (47.5)		
Fetal anomaly					1.570	0.010
No	4222 (99.4)	1593 (99.4)	984 (98.9)	147745 (99.6)		
Yes	25 (0.6)	9 (0.6)	11 (1.1)	667 (0.4)		

Note: GH: gestational hypertension; PE: preeclampsia; SPE: severe preeclampsia; HDP: Hypertensive disorders of pregnancy.

### 3.4 Multiple logistic regression analysis of influencing factors for GH, PE, and SPE

Multiple logistic regression analysis identified several risk factors for GH, PE, and SPE, including maternal age (OR_GH_ = 1.158 (95 % CI: 1.035–1.295), 1.697 (95 % CI: 1.478–1.948), and 2.769 (95 % CI: 2.234–3.433), OR_PE_ = 1.526 (95 % CI: 1.224–1.902) to 2.506 (95 % CI: 1.796–3.497)), non-local residence (OR = 1.124 (95 % CI: 1.018–1.241), 1.212 (95 % CI: 1.032–1.423), and 1.222 (95 % CI: 1.010–1.479)), *in vitro* fertilization (OR = 1.263 (95 % CI: 1.028–1.551), 1.342 (95 % CI: 1.042–1.729) and 1.692 (95 % CI: 1.234–2.321)), graviditas ≤1 (OR_GH_ = 1.126 (95 % CI: 1.035–1.226)), parity <1 (OR_GH_ = 1.140 (95 % CI: 1.026–1.265), OR_PE_ = 1.336 (95 % CI: 1.130–1.581)), non-vaginal delivery (OR_GH_ = 1.525 (95 % CI: 1.223–1.901), 1.185 (95 % CI: 1.058–1.328) an 1.753 (95 % CI: 1.612–1.908); OR_pe_ = 2.108 (95 % CI: 1.428–3.112), 1.464 (95 % CI: 1.174–1.826) and 4.205 (95 % CI: 3.643–4.854), and OR_spe_ = 7.000 (95 % CI: 3.517–13.930), 1.914 (95 % CI: 1.217–3.010) and 19.981 (95 % CI: 14.936–26.730)), maternal hospitalization >7 days (OR = 2.098 (95 % CI: 1.921–2.291), 2.115 (95 % CI: 1.857–2.409), and 1.553 (95 % CI: 1.333–1.810)), and winter birth (OR_GH_ = 1.297 (95 % CI: 1.196–1.408), OR_PE_ = 1.425 (95 % CI: 1.242–1.636)).

In addition, we identified several protective factors for GH, PE, and SPE, including a history of uterine scarring (OR = 0.688 (95 % CI: 0.613–0.773), 0.442 (95 % CI: 0.367–0.533) and 0.268 (95 % CI: 0.215–0.334)), multiple pregnancy (OR_GH_ = 0.679 (95 % CI: 0.518–0.889) OR_SPE_ = 0.298 (95 % CI: 0.223–0.398)), complications of pregnancy (OR = 0.916 (95 % CI: 0.859–0.977), 0.803 (95 % CI: 0.722–0.893) and 0.524 (95 % CI: 0.455–0.603)), hospital of delivery (OR_GH_ = 0.679 (95 % CI: 0.617–0.748), OR_SPE_ = 0.536 (95 % CI: 0.446–0.645)), summer birth (OR_GH_ = 0.670 (95 % CI: 0.609–0.736); OR_PE_ = 0.820 (95 % CI: 0.702–0.958)) and autumn birth (OR_GH_ = 0.773 (95 % CI: 0.706–0.845)). Since 2012, the probability of PE occurrence increased more significantly than that of GH and SPE (OR = 4.612 to 16.657 vs. OR = 0.772 to 0.977 and OR = 1.589 to 2.348). Pregnancy outcomes with a delivery at <37 weeks (OR_PE_ = 1.402 (95 % CI: 1.158–1.698), OR_SPE_ = 2.913 (95 % CI: 2.385–3.558)), low birth weight (OR = 1.508 (95 % CI: 1.265–1.798), 2.438 (95 % CI: 1.976–3.008), and 5.274 (95 % CI: 4.272–6.511)), macrosomia (OR_GH_ = 1.021, PE, 1.407), and a birth length <50 cm (OR = 1.132 (95 % CI: 1.023–1.252), 1.268 (95 % CI: 1.085–1.480), and 1.696 (95 % CI: 1.413–2.035)) were increased due to the occurrence of related HD. Delivery <37 weeks (OR_GH_ = 0.754 (95 % CI: 0.644–0.882)) and >41 weeks (OR = 0.464 (95 % CI: 0.393–0.549), 0.339 (95 % CI: 0.245–0.468) and 0.355 (95 % CI: 0.218–0.578)) were decreased due to the occurrence of HDP. However, there was no significant difference between the two groups in terms of gender and fetal abnormalities following the occurrence of GH, PE, and SPE (all *P* > 0.05), see Table 3.

**Table 3:** Multivariate logistic regression analysis of maternal characteristics and pregnancy outcomes.

Indicators	GH			PE			SPE		
	OR	95 % CI for OR	*P value*	OR	95 % CI for OR	*P value*	OR	95 % CI for OR	*P value*
Maternal age group (years)									
20–24.9	1.315	0.994–1.738	0.055	1.291	0.810–2.060	0.283	1.513	0.912–2.512	0.109
25–29.9	0.982	0.887–1.087	0.723	0.919	0.780–1.082	0.310	0.836	0.684–1.023	0.082
30–34.9	1.158	1.035–1.295	0.010	0.966	0.807–1.157	0.710	0.992	0.795–1.239	0.945
35–39.9	1.697	1.478–1.948	<0.001	1.526	1.224–1.902	<0.001	1.244	0.946–1.637	0.118
≥40	2.769	2.234–3.433	<0.001	2.506	1.796–3.497	<0.001	1.509	0.965–2.360	0.071
<20^a^									
Residence									
non-local	1.124	1.018–1.241	0.021	1.212	1.032–1.423	0.019	1.222	1.010–1.479	0.040
local^a^									
Uterine scar									
Yes	0.688	0.613–0.773	<0.001	0.442	0.367–0.533	<0.001	0.268	0.215–0.334	<0.001
No^a^									
Multiple pregnancy									
Yes	0.679	0.518–0.889	0.005	0.878	0.674–1.144	0.335	0.298	0.223–0.398	<0.001
No^a^									
*In vitro* fertilization									
Yes	1.263	1.028–1.551	0.026	1.342	1.042–1.729	0.023	1.692	1.234–2.321	0.001
No^a^									
Complication of pregnancy									
Yes	0.916	0.859–0.977	0.008	0.803	0.722–0.893	<0.001	0.524	0.455–0.603	<0.001
No^a^									
Graviditas									
No (≤1)	1.126	1.035–1.226	0.006	1.055	0.928–1.200	0.414	1.017	0.860–1.202	0.848
Yes (≥2)^a^									
Parity									
No (<1)	1.140	1.026–1.265	0.014	1.336	1.130–1.581	0.001	0.995	0.811–1.220	0.961
Yes (≥1)^a^									
Hospital of delivery									
hospital 1	1.083	0.956–1.228	0.210	1.071	0.873–1.313	0.510	0.898	0.705–1.142	0.380
hospital 2	0.679	0.617–0.748	<0.001	0.955	0.817–1.117	0.567	0.536	0.446–0.645	<0.001
hospital 3^a^									
Mode of delivery									
Forceps delivery	1.525	1.223–1.901	<0.001	2.108	1.428–3.112	<0.001	7.000	3.517–13.930	<0.001
Lateral vaginal incision	1.185	1.058–1.328	0.003	1.464	1.174–1.826	0.001	1.914	1.217–3.010	0.005
Anterior vaginal incision	1.093	0.914–1.308	0.328	0.792	0.529–1.187	0.258	1.734	0.742–4.056	0.204
Cesarean section	1.753	1.612–1.908	<0.001	4.205	3.643–4.854	<0.001	19.981	14.936–26.730	<0.001
Vaginal delivery^a^									
Maternal hospitalization time (days)									
>7	2.098	1.921–2.291	<0.001	2.115	1.857–2.409	<0.001	1.553	1.333–1.810	<0.001
≤7^a^									
Birth Year									
2013	0.772	0.642–0.928	0.006	4.612	2.338–9.099	<0.001	1.589	1.051–2.402	0.028
2014	0.706	0.590–0.845	<0.001	7.407	3.852–14.246	<0.001	1.758	1.174–2.633	0.006
2015	0.661	0.549–0.797	<0.001	8.624	4.480–16.601	<0.001	2.348	1.568–3.516	<0.001
2016	0.617	0.520–0.731	<0.001	7.706	4.037–14.709	<0.001	1.704	1.147–2.532	0.008
2017	0.631	0.534–0.745	<0.001	7.107	3.726–13.554	<0.001	1.603	1.081–2.378	0.019
2018	0.632	0.534–0.748	<0.001	7.695	4.032–14.686	<0.001	1.823	1.227–2.709	0.003
2019	0.799	0.679–0.941	0.007	13.607	7.189–25.753	<0.001	1.426	0.951–2.138	0.086
2020	1.059	0.902–1.244	0.484	16.354	8.644–30.938	<0.001	1.410	0.932–2.135	0.104
2021	0.977	0.831–1.150	0.783	16.657	8.802–31.524	<0.001	1.585	1.050–2.393	0.028
2012^a^									
Birth season									
Summer	0.670	0.609–0.736	<0.001	0.820	0.702–0.958	0.012	0.986	0.813–1.196	0.886
Autumn	0.773	0.706–0.845	<0.001	1.042	0.901–1.206	0.581	1.132	0.940–1.364	0.190
Winter	1.297	1.196–1.408	<0.001	1.425	1.242–1.636	<0.001	1.175	0.973–1.419	0.095
Spring^a^									
Gestational age (weeks)									
<37	0.754	0.644–0.882	<0.001	1.402	1.158–1.698	0.001	2.913	2.385–3.558	<0.001
>41	0.464	0.393–0.549	<0.001	0.339	0.245–0.468	<0.001	0.355	0.218–0.578	<0.001
37–41^a^									
Newborn gender									
male	1.008	0.948–1.072	0.798	1.046	0.946–1.157	0.379	0.976	0.857–1.112	0.716
female^a^									
Birth weight (g)									
<2500	1.508	1.265–1.798	<0.001	2.438	1.976–3.008	<0.001	5.274	4.272–6.511	<0.001
>4000	1.021	0.878–1.188	0.786	1.407	1.133–1.748	0.002	1.029	0.715–1.482	0.877
2500–4000^a^									
Birth length (cm)									
<50	1.132	1.023–1.252	0.016	1.268	1.085–1.480	0.003	1.696	1.413–2.035	<0.001
≥50^a^									
Fetal anomaly									
Yes	1.091	0.728–1.634	0.673	0.739	0.378–1.443	0.375	1.064	0.564–2.008	0.848
No^a^									

Note: GH: gestational hypertension; PE: preeclampsia; SPE: severe preeclampsia.

a Reference.

## 4 Discussion

In this study, we found that the total incidence of HDP in Hangzhou, China was 45.17 ‰; specifically, the incidence of GH, PE, SPE, eclampsia, HELLP syndrome, chronic hypertension, chronic hypertension combined with PE or SPE, and postpartum eclampsia were as follows: 27.32 ‰, 10.31 ‰, 6.40 ‰, 0.06 ‰, 0.28 ‰, 0.28 ‰, 0.50 ‰ and 0.03 ‰. HDP and the incidence of various subtypes showed a rising trend annually. The incidence of HDP varies from place to place, possibly due to differences in the definition of HDP, population composition, demography, and maternal characteristics. A previous meta-analysis showed that the prevalence of HDP, GH, PE, mild preeclampsia, SPE, eclampsia, chronic hypertension, and chronic hypertension combined with PE, in China from 1990 to 2020 were 7.30 %, 3.30 %, 4.50 %, 2.00 %, 2.60 %, 0.11 %, 0.60 %, and 0.60 %, respectively, and were higher than our present findings [19]. These data indicate that the incidence of HDP in Hangzhou is significantly lower than the national level in China. However, the total incidence of HDP in this study was slightly higher than that in India (4.40 %) [20]. However, the results of our present study are lower than the prevalence of HDP in non-metropolitan areas and metropolitan areas in the United States (7.40 % and 6.60 %), as reported by Kloppenburg et al. [21]. In the present study, we found that HDP increased on an annual basis after 2015; a previous study also reported that the incidence of HDP doubled from 6.00 % in 2000 to 12.00 % in 2018 [22]. Furthermore, our analyses showed that the incidence of HDP initially decreased from 2012 to 2015, and then increased annually after 2015. Similarly, between 2011 and 2019, the rate of HDP for first-time single live births in the United States increased by 7.30 % per year, from 57.20 % in 2011 to 99.70 % in 2019 [23].

In this study, we identified several risk factors for HDP, including few parity or no gravidity, AMA, non-local residence, *in vitro* conception, increased maternal hospital stay, and winter birth. A history of uterine scarring, multiple pregnancy, pregnancy complications, hospital of delivery, and summer and autumn births, were all identified as protective factors for HDP. Since 2012, the of PE occurrence increased more significantly than that of GH and SPE. The outcomes of delivery at <37 weeks, a low birth weight, macrosomia, and a birth length <50 cm all increased due to the occurrence of HDP. The occurrence of GH reduced the risk of delivery at <37 weeks and the occurrence of HDP reduced the risk of >41 gestation weeks. Maducolil et al. showed that compared with women with normal blood pressure, PE was significantly associated with a higher probability of cesarean section (OR = 2.670), acute maternal morbidity (OR = 16.420), stillbirth (OR = 3.270), preterm birth (OR = 8.670), and a higher risk of premature birth (OR = 8.670), NICU admission rates (OR = 4.410) and low birth weight (OR = 7.930). Furthermore, women with PE were older, childless, had diabetes and obesity, and had an increased risk of preterm birth and delivery by cesarean section [10]. In another study, Li et al. reported that AMA, assisted reproductive technology, living in rural areas, a history of HDP, male fetus, multiple pregnancy, and PE patients with three or more risk factors, such as polycystic ovary syndrome, hemolysis, elevated liver enzymes and low platelet count syndrome, intrahepatic cholestasis of pregnancy, cardiovascular disease, gestational diabetes, systemic lupus erythematosus, thyroid disease, or liver disease, had an increased risk of serious adverse outcomes [24]. Chronic hypertension, systemic lupus erythematosus, pregestational diabetes, twin pregnancy, AMA, and fertility treatment, were found to be the strongest independent risk factors. Although the incidence of twin pregnancy and pregestational diabetes increased in a linear manner, fertility treatment decreased linearly [25]. Das et al. suggested that maternal age, first birth, early pregnancy, twin pregnancy, chronic hypertension, urinary tract infection, and gestational diabetes, were important risk factors for PE [26].

In Table 3, we showed that the risk of pregnant women ≥30 years with GH increased, OR = 1.158 increased to 2.769 at 40 years old, PE pregnant women ≥35 years old increased, OR = 1.526 increased to 2.506 at 40 years old, but the age change of SPE pregnant women was not associated with the disease. In Ireland, the age at which mothers give birth is increasing, and the association of hypertension with increasing age will undoubtedly lead to an increase in HDP and its incidence, with potentially adverse consequences for pregnant women and their babies [27]. Li et al. also showed that AMA will increase the risk of PE and GH, but the size of the impact is different, while women ≤25 years old or with low body mass index in early pregnancy have a significantly reduced risk of GH [28]. A higher incidence of adverse fetal outcomes was observed in women with HDP, especially those <20 years of age or >35 years of age or those diagnosed with superimposed PE. More attention should be paid to cases of PE plus chronic hypertension and pregnant women <20 or >35 years of age to reduce the burden of adverse fetal outcomes due to HDP [29].

This study shows that non-local residence is a risk factor for GH, PE, and SPE, and Mattsson believes that women with lower socioeconomic status have an increased risk of PE, even in a government-funded universal healthcare environment [30]. A 15-year retrospective study at the University of Maiduguri Teaching Hospital in Maiduguri, Nigeria, showed that no formal education, unemployment, coma for 10 h or more, vaginal delivery and severe hypertension, unregistered status, and multiple pregnancy were significantly associated with adverse maternal and infant outcomes [31].

Teka et al. showed that women with preeclampsia-eclampsia syndrome, suffering from early-onset diseases, had higher levels of adverse maternal outcomes [32]. There is also a significant increase in perinatal morbidity and mortality among women with early-onset diseases. Prolonged maternal hospital stay (aOR = 4.700) was similar to the risk factors for GH, PE, and SPE shown in this study (OR = 2.098, 2.115, and 1.553).

GH, PE, and SPE were associated with increased risk of pregnancy outcomes in different birth years, winter birth, gestation weeks <37, low birth weight, macrosomia, and length <50 cm. The occurrence of GH, PE, and SPE was associated with a reduced risk of summer and fall births and delivery at >41 weeks. Li et al. [28] showed that maternal age over 35, body mass index over 24, and pregnancy complicated with gestational diabetes would increase the risk of PE and GH, but the impact was different. Primary birth and winter and spring births (compared to summer births) were risk factors for GH only. Pregnancy combined with diabetes/kidney disease/heart disease are the only risk factors for PE. Women who are younger than 25 years of age or have a low body mass index in early pregnancy have a significantly reduced risk of developing GH. Mothers with male fetuses are more likely to develop PE, indicating that the risk factors for GH and PE are not exactly the same, and behind these differences may be their different etiologies and mechanisms. Shayan showed that autumn fertilization (OR = 1.130) may increase the risk of PE, and AMA, low educational background, blood type O, and fertilization in the cold season may be risk factors for PE [33]. Maternal GH and PE are risk factors for respiratory diseases in full-term and preterm newborns [34]. PE is significantly associated with maternal and neonatal morbidity and mortality and increased vaginal surgical delivery, cesarean section, LBW, and birth asphyxia [35].

Although we conducted a multi-center and large-sample study, the data in this study lacked information such as height, weight, and blood pressure of pregnant women, and sub-class analysis of variables such as height, weight, and blood pressure could not be conducted, which may cause some bias in the results. As there were only 68 pregnant women with less than 28 weeks of gestation age in this data, the number of cases was too small compared with 160,033. Many studies also excluded pregnant women with less than 28 weeks of gestation age, so the exclusion of pregnant women with less than 28 weeks of gestation age may lead to the lack of research on abortion caused by HDP. This is the limitation of this study, and it is necessary to strengthen the collection of such information in the later stage to further improve the results.

## 5 Conclusion

The incidence of HDP and its subtypes shows an overall increasing trend from 2012 to 2021. Maternal factors and pregnancy outcomes may be strongly associated with HDP. Future studies should focus more on how to reduce the incidence of HDP by controlling maternal factors to ultimately reduce the incidence of adverse pregnancy outcomes.

## CRediT authorship contribution statement

**Yiming Chen:** Writing – review & editing, Writing – original draft. **Bin Wu:** Writing – original draft. **Huimin Zhang:** Data curation. **Xuelian Chu:** Resources, Data curation. **Lingling Huang:** Methodology. **Yanan Wang:** Investigation. **Xiufeng Liang:** Project administration.

## Data sharing statement

Data are available on request to the corresponding author.

## Ethics approval and consent to participate

The study has been conducted under the approval of the Human Research Ethics Committee of the Hangzhou Hospital ([2023] Medical Ethics Review A (013), and the procedures have been performed by the Declaration of Helsinki. Since this study is a retrospective study, the need to obtain informed consent was waived by the Human Research Ethics Committee of the Hangzhou Women's Hospital.

## Funding

This work was supported by the Zhejiang Medicine and Health Scientific Research Project (2021KY258).

## Declaration of competing interest

The authors declare that they have no known competing financial interests or personal relationships that could have appeared to influence the work reported in this paper.
